# Subcapsular renal hematoma after ureterorenoscopy: An unknown complication of a known procedure

**DOI:** 10.4103/0974-7796.68861

**Published:** 2010

**Authors:** Ujjwal Bansal, Ajit Sawant, Jayesh Dhabalia

**Affiliations:** Department of Urology, LTMG Hospital, Sion, Mumbai, India

**Keywords:** Percutaneous drain, subcapsular renal hematoma, ureterorenoscopy

## Abstract

Renal subcapsular hematoma is not an uncommon complication after extracorporeal short wave lithotripsy, trauma, renal angiographic procedures and spontaneously in patients of malignancy and in patients on anticoagulation. We present a patient who developed renal subcapsular hematoma after ureterorenoscopy, which has not been mentioned in literature ever. Clinical spectrum varies from spontaneous resolution through acute renal failure to Page kidney. Page kidney is the external compression of a kidney usually caused by a subcapsular hematoma associated with high blood pressure and occasional renal failure. It is named after Dr. Irvin Page who first demonstrated in 1939 that wrapping cellophane tightly around animal kidneys could cause hypertension. Various management options are mentioned in literature and depend upon the severity of hematoma. Percutaneous drainage is a successful option for the management of subcapsular hematoma in hemodynamic stable patients.

## INTRODUCTION

Renal subcapsular hematoma is not an uncommon complication after extracorporeal short wave lithotripsy (ESWL), trauma, renal angiographic procedures and spontaneously in patients of malignanacy and in patients on anticoagulation.[1,2] We present a patient who developed renal subcapsular hematoma after ureterorenoscopy (URS), which has not been mentioned in literature ever.

## CASE REPORT

**Figure 1:**
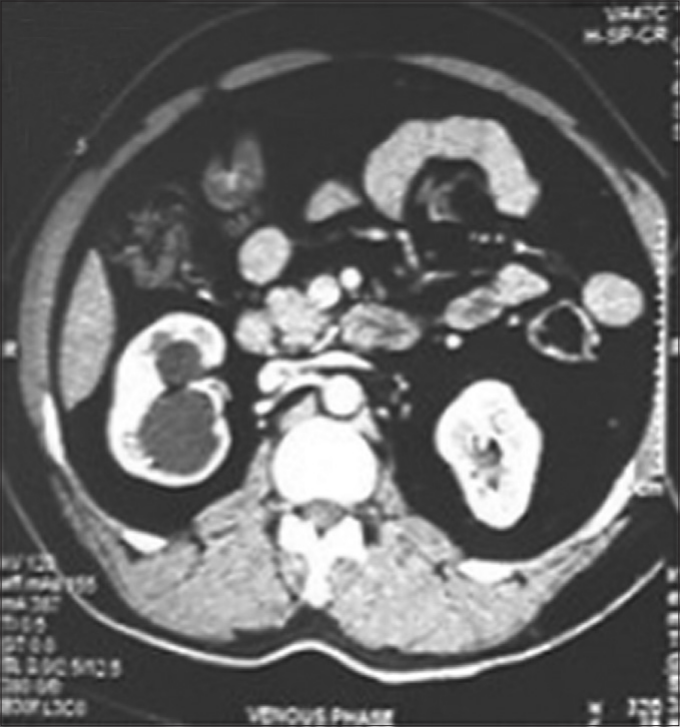
Preoperative CT scan showing normal renal parenchyma

A 35-year-old male patient, a known case of controlled diabetes mellitus on insulin, presented with a right lower ureteric radiolucent calculus (1.0×0.7 cm) with moderate dilatation of pelvicalyceal system (PCS), diagnosed on Ultrasonography (USG) and confirmed on contrast-enhanced computed tomography (CECT) scan with good renal parenchyma and function on computarized tomography (CT) scan [Figure 1].

The patient underwent URS procedure; 7.8/9 Fr. semi rigid URS was introduced. Right ureteric orifice was dilated by 4 mm, 5 cm and 25 atmosphere pressure balloon dilator. Right ureteric catheter 6 Fr., followed by 0.032 inch J tip angiographic guide wire, introduced under fluoroscopic guidance, across the stone into PCS. URS was introduced, stone was visualized in lower ureter and was blasted by pneumatic lithoclast. Retrograde 5 Fr. 26-cm double J stent was kept. The rest of the procedure was uneventful.

**Figure 2:**
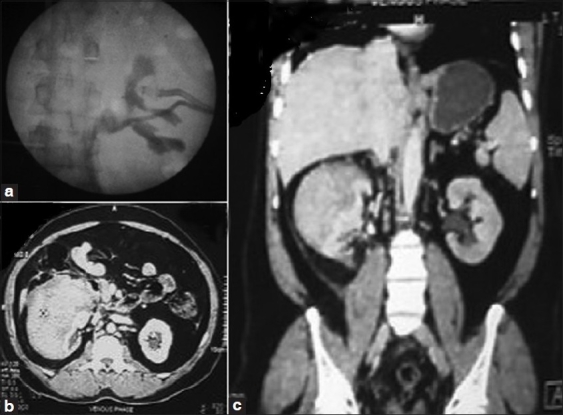
(a) Retrograde pyelogram (RGP) showing stretched out Pelvicalyceal system (PCS) with drain *insitu* in hematoma. (b,c) CT images showing subcapsular hematoma with compressed and displaced right renal parenchyma

Postoperatively 12 hours later, the patient developed severe pain in right flank with severe tenderness. USG was suggestive of 11×7 cm sized perirenal hematoma with DJ stent *in situ* and mild dilatation of PCS. The patient was managed conservatively, but 24 hours later he developed fever. CECT was suggestive of large nonenhancing perirenal hematoma of size 11×12×10 cm (HU 60). The right kidney appeared to be pushed medially, downward and seemed to be collapsed. It showed poor excretion of contrast [Figure 2].

The patient was not responding to conservative management with recurrent fever and persistent pain, so decision was taken to intervene. Retropyelogram was suggestive of extravasation of contrast with stretched out PCS [Figure 2]. Pigtail 8 Fr. percutaneous (PCN) catheter was inserted in view of extravasation. A Malecot’s catheter 10 Fr. was inserted into hematoma for drainage.

Post procedure pain and fever were relieved. Follow-up USG (fifth day) was showing reduced hematoma of size up to 9×9×8 cm. Malecot’s catheter was removed 5 days after confirmation on nephrostomogram of no leak. Subsequently, PCN was removed 2 days later. Patient was discharged with advice to repeat CECT after a month.

## DISCUSSION

The subcapsular area of the kidney is a potential space where fluid can accumulate, causing compression of the renal parenchyma.[1] Subcapsular renal hematomas are usually related to kidney trauma and are well-known complications of ESWL.[2] The causes of spontaneous hematoma include tumors, vascular diseases, infections, cystic diseases, hydronephrosis, preeclampsia and blood dyscrasias.[3] Subcapsular hematoma postureteroscopic intracorporeal lithotripsy is not reported in literature.

In this paper we discuss our experience with a case of post-URS subcapsular/perirenal hematoma. Subcapsular hematoma in previous enumerated etiologies is due to trauma to renal vessels or renal parenchyma. In our patient, there was no obvious trauma to PCS or renal parenchyma and operative procedure was uneventful. The most probable explanatation for development of hematoma could be trauma to PCS during guide wire manipulation or raised intrarenal pressure leading to forniceal rupture, separation of capsule from parenchyma and hematoma. Probable etiology of hematoma can be explained by injury due to guide wire while access during ureteroscopy.

The clinical presentation of these patients varies considerably based on the degree and duration of the bleeding. Acute onset of flank or abdominal pain is the most common symptom. Other patients may present with hematuria, a palpable mass or signs of blood loss.[3]

Treatment of renal subcapsular hematomas has been debated. We managed this patient by pigtailing of hematoma and percutaneous nephrostomy in view of severe persistent fever and pain. As per the literature, small, asymptomatic hematomas resolve rapidly and spontaneously and are usually managed by conservative management. Conservative management includes antibiotics, control of pain with monitoring of vital signs, serum creatinine and Hb values.[2]

Indications for early intervention include unbearable pain, infective complications, unstable vitals due to uncontrolled bleeding, and renal compression and ischemia with non-viable renal parenchyma. In large hematomas, evacuation should be considered for prevention of kidney function impairment and a potential secondary hypertension and even to shorten resolution.[2] Function of the kidney suffering hematoma may be compromised to a greater or lesser extent after the hematoma occurs but with a normal contralateral kidney, there will be no significant laboratory changes in the overall kidney function.[2] However, bilateral hematoma may lead to anuria. After putting pigtail for subcapsular hematoma, the patient responded well and fever and pain subsided. There was no drain output from pigtail and also USG showed resolution in size of hematoma so decision was taken to remove the pigtail. Various minimally invasive approaches for drainage have been described in literature.

A percutaneous drainage, laparoscopic decortication may be done to decrease surgical aggressiveness in patients with stable vitals but with unbearable pain or renal compression.[2,4] If hematoma is rapidly progressive or the vitals are deteriorating, conventional drainage by open surgery using lumbotomy may be performed.[2]

We have preferred percutaneous pigtailing in this case as open surgery, during active bleeding, usually ends in nephrectomy unfortunately.

In view of Héctor Pastor Navarro *et al*., once the immediate acute condition has been resolved and the patient and the size of the hematoma are stable, hematoma should be evacuated as a delayed emergency procedure 8–15 after its occurrence, when active bleeding would have stopped and no fibrosis would have occurred yet.[2]

This patient responded well to our treatment and a follow-up of 2 weeks was uneventful. The patient was called up for follow-up CT scan after a month.

## CONCLUSION

Subcapsular renal hematoma is a well-known complication of ESWL, renal angiographic procedures, post-traumatic and spontaneously after anticoagulation. However, it can also occur after subtle routine procedures like URS.
